# Multi-omic profiling reveals associations between the gut microbiome, host genome and transcriptome in patients with colorectal cancer

**DOI:** 10.1186/s12967-024-04984-4

**Published:** 2024-02-18

**Authors:** Shaomin Zou, Chao Yang, Jieping Zhang, Dan Zhong, Manqi Meng, Lu Zhang, Honglei Chen, Lekun Fang

**Affiliations:** 1 Department of General Surgery, The Sixth Affiliated Hospital, Sun Yat-sen University, Guangzhou, 510655 China; 2 Guangdong Provincial Key Laboratory of Colorectal and Pelvic Floor Disease, The Sixth Affiliated Hospital, Sun Yat-sen University, Guangzhou, 510655 China; 3 Key Laboratory of Human Microbiome and Chronic Diseases (Sun Yat-sen University), Ministry of Education, Guangzhou, 510655 China; 4 Biomedical Innovation Center, The Sixth Affiliated Hospital, Sun Yat-Sen University, Guangzhou, 510655 China; 5 Department of Computer Science, Faculty of Science, Hong Kong Baptist University, Hong Kong, China; 6 Gastrointestinal Endoscopy Center, The Eighth Affiliated Hospital, Sun Yat-sen University, Shenzhen, 518033 China

**Keywords:** Colorectal cancer, Gut microbiota, Metagenomic sequencing, Somatic mutations, Transcriptome, Metabolism, Immune response

## Abstract

**Background:**

Colorectal cancer (CRC) is the leading cancer worldwide. Microbial agents have been considered to contribute to the pathogenesis of different disease. But the underlying relevance between CRC and microbiota remain unclear.

**Methods:**

We dissected the fecal microbiome structure and genomic and transcriptomic profiles of matched tumor and normal mucosa tissues from 41 CRC patients. Of which, the relationship between CRC-associated bacterial taxa and their significantly correlated somatic mutated gene was investigated by exome sequencing technology. Differentially expressed functional genes in CRC were clustered according to their correlation with differentially abundant species, following by annotation with DAVID. The composition of immune and stromal cell types was identified by XCELL.

**Results:**

We identified a set of 22 microbial gut species associated with CRC and estimate the relative abundance of KEGG ontology categories. Next, the interactions between CRC-related gut microbes and clinical phenotypes were evaluated. 4 significantly mutated gene: TP53, APC, KRAS, SMAD4 were pointed out and the associations with cancer related microbes were identified. Among them, *Fusobacterium nucleatum* positively corelated with different host metabolic pathways. Finally, we revealed that *Fusobacterium nucleatum* modified the tumor immune environment by TNFSF9 gene expression.

**Conclusion:**

Collectively, our multi-omics data could help identify novel biomarkers to inform clinical decision-making in the detection and diagnosis of CRC.

**Supplementary Information:**

The online version contains supplementary material available at 10.1186/s12967-024-04984-4.

## Introduction

Colorectal cancer (CRC) is highly aggressive and ranks as the third leading malignancy in the world population, causing nearly 500,000 deaths per year. The incidence of CRC remains a health care challenge worldwide [1–3]. Therefore, there is an urgent need to characterize biomarkers for CRC**.**

Advances in metagenome-wide association studies of fecal samples have identified microbial markers of CRC, and the causal effect of bacteria on cancer has been recognized [4–7]. The gut microbiota, containing at least 38 trillion bacteria, is critical for the maintenance of homeostasis and health, including the digestion of food, vitamin biosynthesis, behavioral responses, and protection from pathogens. Emerging evidence has shown that the dysbiosis of gut microbiota can lead to alterations in host physiology, resulting in the pathogenesis of CRC [4, 8].

The interplay between microorganisms and the host immune system frequently occurs in the gastrointestinal tract [9]. Eleven bacterial strains that induce IFNγ + CD8 T cells, including *Eubacterium limosum*, have been isolated, and these strains can enhance host resistance to *Listeria monocytogenes* and increase the efficacy of immune checkpoint inhibitor therapy [10]. On the other hands, the microbiome also impacts intestinal inflammation, a hallmark of the neoplastic transformation of epithelial cells, which thereby furthers CRC development [11]. For instance, *Fusobacterium nucleatum* can activate TLR4 signaling to NFκB which facilitates tumorigenesis [12]. However, the detailed mechanisms mediating host–microbiota interactions in CRC remain unclear. Few studies have addressed the relationship between human intestinal microbiota and tumor gene expression profiling during tumorigenesis. The limitations may be due to the difficulty in obtaining microbiota and tumor samples from the same cohort for analysis.

In this study, stool and tissue samples were collected from a cohort of 41 CRC patients. We performed the high-throughput profiling of bacterial communities and figured out its interlink with the genomic landscape and transcriptome of CRC. Cancer associated microbiome alterations were firstly identified and their correlations with clinical covariates were then discussed. Also, we identified frequently mutated genes and investigated their effects on microbiome structure and function. The dynamic changes in microbiota composition and tumor gene expression were further compared. Finally, the interplay between differentially abundant species and immune and metabolic pathways were explored to uncover more important factors for gut homeostasis.

## Materials and methods

### Sample collection

We obtained snap-frozen tissue samples from a cohort of 41 CRC patients who underwent curative resection at the Sixth Affiliated Hospital of Sun Yat-sen University with patients’ written informed consent and approval. Stool samples from the same cohort of 41 CRC patients were collected and stored at − 20 °C within 4 h and subsequently − 80 °C within 24 h. None of the patients had taken antibiotics within 2 months or received preoperative chemotherapy or radiotherapy prior to sample collection.

### Metagenomic DNA sequencing and analysis

Microbial DNA was extracted from stool samples (200 mg) by the phenol/chloroform/isoamyl alcohol method. Qualified fecal genomic DNA was extracted to construct libraries using a TruSeq DNA HT Sample Prep Kit and then subjected to sequencing on the Illumina platform (paired-end 150 bp). The raw sequencing data were filtered with SOAPnuke to remove the low-quality reads and adapter contamination. Subsequently, host (human) contamination was removed by aligning reads to the human genome with SOAP2 [13] After sequence quality control, we employed MetaPhlAn2 [14] to align the high-quality reads to the clade-specific marker genes and calculate the taxonomic relative abundance profile. To identify disease associated biomarkers, we conducted the LEfSe [15] analysis on taxonomy relative abundance using the parameters “-w 0.05 -l 2.0”. Functional changes were estimated using HUManN2 [16] with a customized KEGG database. Differentially abundant KEGG pathways were determined using the previously described reporter scores [17].

### Exome DNA sequencing and analysis

Human genomic DNA was extracted from both tumor and adjacent normal tissues. The qualified genomic DNAs were randomly fragmented by Covaris Ultrasonicator, and then ligated to Illumina sequenced adapters. DNA fragments with length ranging from 350 to 500 bp were extracted, amplified by ligation-mediated PCR (LM-PCR), purified, and subsequently hybridized to the NimbleGen SeqCap EZ Exome (44 M) array for enrichment. The captured libraries were then sequenced on the Illumina HiSeq X Ten platform, generating 150-bp paired-end reads. After DNA sequencing, SOAPnuke was utilized to remove low-quality sequence and adapter contamination from the raw reads. The clean sequencing reads were aligned to the human reference genome with the Burrows-Wheeler Aligner (BWA) [18]. SAMtools [19] was employed to mark and remove PCR duplicates. For somatic mutations, we used MuTect [20] to detect somatic SNVs, with a minimum depth requirments of 20 × for both normal and tumor samples. Somatic INDELs were called using the Somatic Indel Detector command from Genome Analysis Toolkit [21] with default parameters. Highly confident INDELs were determined by an in-house pipeline and further classified as either germline or somatic based on the presence of any evidence of the event at the same locus was observed in the normal data. Finally, SNVs and INDELs were combined and annotated with Oncotator [22]. To identify significantly mutated genes, we applied the MutSigCV [23] on the annotated somatic SNVs and INDELs.

### Host RNA sequencing and analysis

We extracted total human RNA from tumor and matched adjacent normal tissues. The human RNA was fragmented and further purified with the RNA Clean XP Kit. Subsequently, these RNAs were qualified using a Nano Drop and Agilent 2100 bioanalyzer. RNA sequencing (RNA-seq) was carried out on the Illumina platform, generating 150 bp paired-end reads. SOAPnuke was used to remove the low-quality reads and reads containing adapters from the raw sequencing data. Subsequently, rRNA contamination was then removed by mapping the reads against the rRNA database with SOAP2 (doi: 10.1093/bioinformatics/btp336). To quantify gene expression, we first aligned the clean sequencing reads to the human reference genome using STAR [24]. HTSEQ [25] was subsequently employed to count the number of reads aligned to each protein-coding genes. We used EBseq [26] to identify differentially expressed genes based on normalized read count data. Gene set enrichment analysis (GSEA) [27] was adopted to assess the pathway alterations, and significant pathways were determined by p values calculated on the basis of hypergeometric distribution with Benjamini correction. xCell [28] was employed to determine the cell-type enrichment scores from the RNA expression data.

### Integrated analysis of microbiome data with somatic alterations and deregulated genes

Based on the taxonomic profiles and functional pathway abundance, we used LEfSe to assess microbial difference between subjects with or without specific somatic mutated genes. The significance was determined by LDA scores with a threshold of 2.0. Furthermore, the correlations between differentially abundant species and deregulated genes were estimated using spearman’s rank test. Associations with P values < 0.01 were considered statistically significant.

## Results

### Identification of a set of gut microbes associated with CRC

**Fig. 1:**
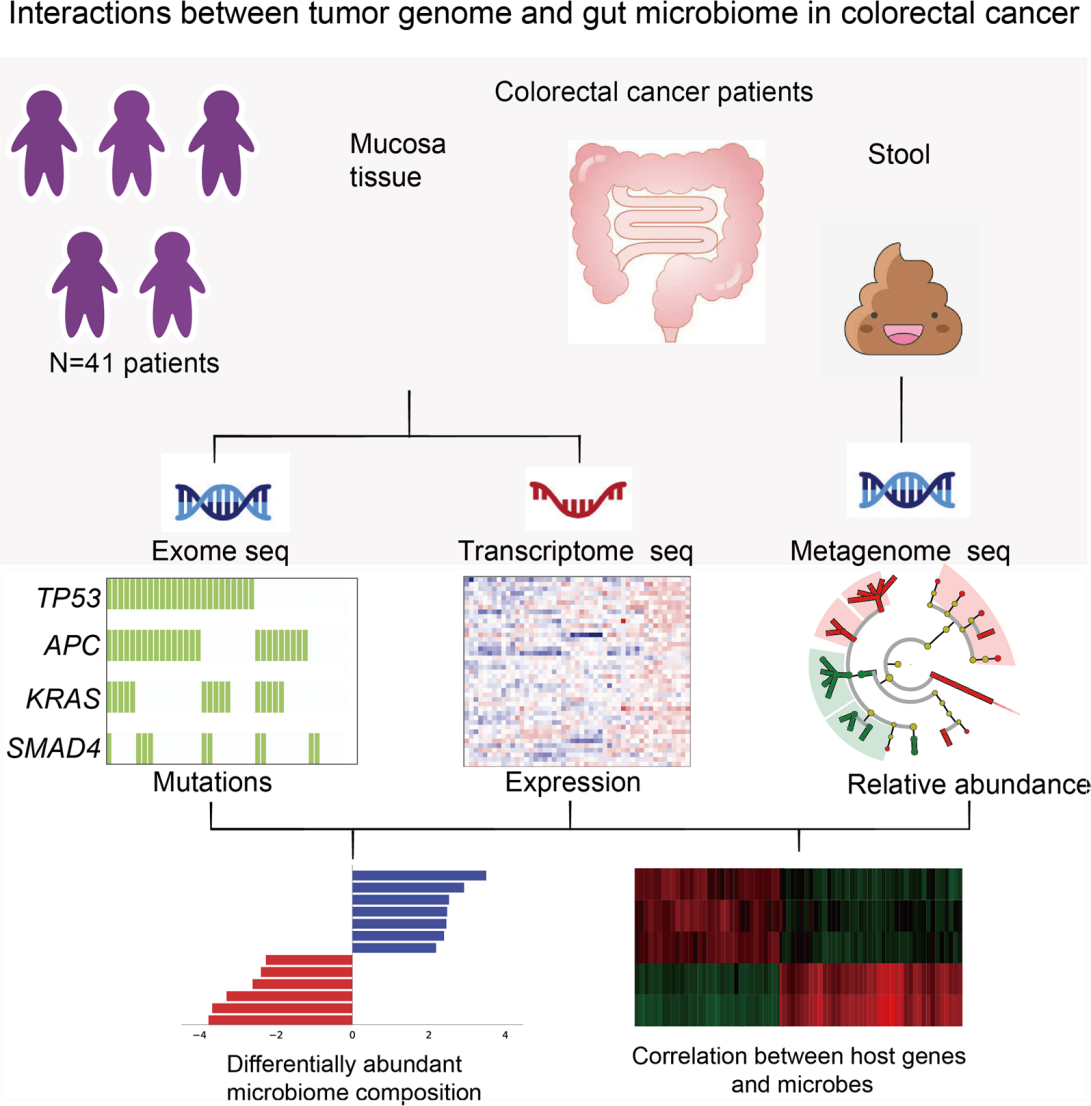
Metagenomics sequencing of the stool sample and exome and transcriptome sequencing of mucosa tissue in colorectal cancer. We collected stool specimens and matched tumor and normal mucosa tissue from 41 colorectal cancer patients. The former samples were metagenomically shotgun sequenced to yield taxonomic and functional profiles; the latter were processed using exome and transcriptome sequencing technology respectively. Features of the microbiome were correlated with clinic elements somatic mutations, and differentially expressed genes, respectively

Most colorectal cancers arise from adenoma to carcinoma as verified by diet, inflammatory processes, gut microbiota, and genetic alterations. Nonetheless, the mechanism by which the microbiota interacts with these etiologic factors to promote CRC is not clear. Therefore, we collected stool samples, tumor and matched normal tissues from 41 CRC individuals, and carried out multi-omics sequencing analyses to evaluate the interplay between cancer cells and gut microbiome (Fig. 1 and Additional file 1: Table S1). As shown in Additional file 1: Fig. S1a, the stool samples were subjected to metagenomic sequencing, achieving an average of 7 Gb clean data. Additionally, we conducted whole exome sequencing, ensuring a minimum of more than 100X coverage and 20 Gb data, respectively (Additional file 1: Table S2).

**Fig. 2:**
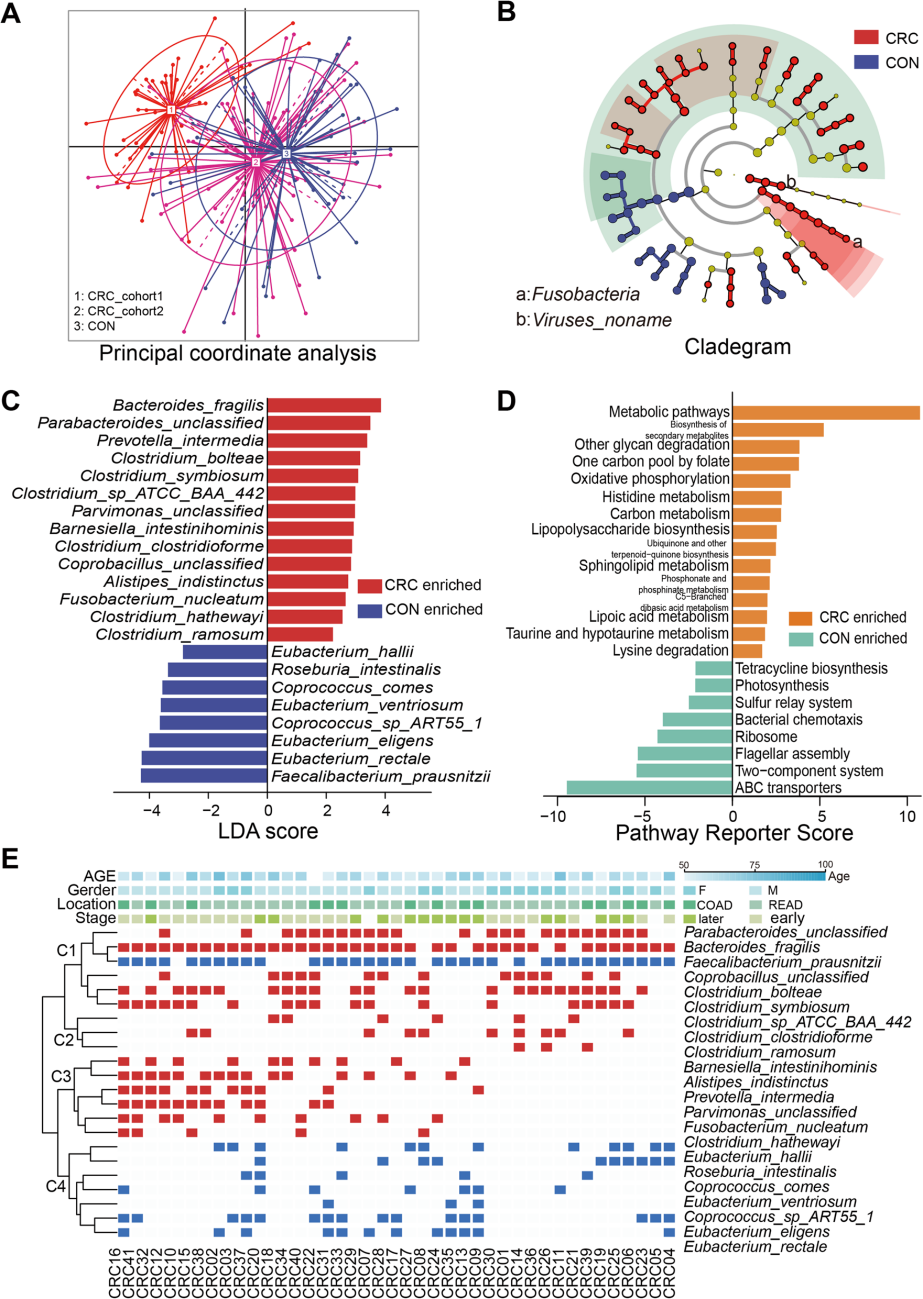
**A** Microbiome alteration between healthy and CRC subjects. PCoA plot showed the two cohorts used in our project. **B** Taxonomic profile difference detected with LEfSe. **C** Differentially abundant species between healthy controls and CRC patients. **D** Differentially abundant KEGG pathways between healthy controls and CRC patients. **E** Unsupervised clustering uncovered associations between differentially abundant species and clinic covariates

We first examined the microbiome dysbiosis by integrating our metagenomic sequencing data with a public Chinese colorectal cancer cohort^3^ (CRC cohort2 and CON) (Fig. 2A). Compared with healthy controls, the CRC patients in our cohort exhibited a significantly decreased alpha diversity (Additional file 1: Fig. S1b), but no obvious difference in the beta diversities (Additional file 1: Fig. S1c). To investigate the alterations in microbiota structure, we conducted the linear discriminant analysis effect size (LEfSe) analysis to compare healthy controls and combined tumor samples. Totally, there were 2 taxa (*Viruses_noname* and *Fusobacteria*) at the phylum level and 10 at the genus level significantly altered respectively (Fig. 2B and Additional file 1: Table S3). Notably, we figured out 22 species associated with disease status, of which 14 were elevated in CRC group (Fig. 2C). Of them, *Bacteroides fragilis* (LDA = 3.897), *Parabacteroides spp.* (LDA = 3.499) and *Prevotella intermedia* (LDA = 3.452) exhibited the highest abundances in CRC patients. In contrast, eight species were enriched in healthy controls, including *Faecalibacterium prausnitzii* (LDA = 4.299), *Eubacterium rectale* (LDA score = 4.255), *Eubacterium eligens* (LDA = 4.002), and so on.

To further investigate the functions of 22 tumor-associated bacteria, we used HUManN2 to estimate the relative abundance of KEGG ontology (KO) categories. Disease associated KEGG pathway changes were further identified using the method described in Feng Q. et al.^4^ We observed that bacteria related metabolic pathways were enriched in CRC groups. Especially, one carbon pool by folate metabolic pathway of microbiota was significantly (Reporter score = 3.471) higher in CRC patients (Fig. 2D). The one carbon pool by folate is a universal cell metabolic process supporting tumorigenesis, obtaining folate (vitamin B9) and cobalamin (vitamin B12) from diet. Furthermore, the cancer enriched species showed positive correlations with the metabolic pathways such as carbon metabolism and oxidative phosphorylation, whereas some well-known beneficial bacteria (including *Faecalibacterium prausnitzii*), displayed negative correlations (Additional file 1: Fig. S2).

### Clinical phenotypes and related microbial taxonomic in CRC

Next, we investigated associations between overall microbiome configuration with CRC clinical covariates. Clinically, of the cohort’s 41 individuals (63% male; ages 46–79), 26 subjects (63%) belong to COAD and 15 subjects (37%) had carcinomas at rectum. Additionally, 10 subjects were diagnosed at early stage and 31 subjects (76%) at later stage. Among all 41 individuals, we observed that several *paraprevotella.ssp* were elevated in patients with age < 65 (for example, *paraprevotella clara,* LDA score = 3.051; *paraprevotella xylaniphila,* LDA score = 2.478) (Additional file 1: Fig. S3a). Furthermore, *Clostridium clostridioforme* was predominated found in females (Additional file 1: Fig. S3b**,** LDA score = 3.182). As to *Bacteroides* genus, the abundance of *Bacteroides eggerthii* was significantly increased in COAD (LDA score = 3.625) whereas *Bacteroides massiliensis* was enriched in READ (LDA score = 4.985) (Additional file 1: Fig. S3c). *Bifidobacterium,* one of the major probiotics, exhibited a significant increase in the early stage and individuals with age < 65 (*Bifidobacterium longum,* LDA score = 3.698; *Bifidobacterium dentium,* LDA score = 2.102) (Additional file 1: Fig. S3a and d).

We also assessed the connections between clinical characteristics and 22 cancer associated bacteria in our subjects through unsupervised clustering (Fig. 2E and Additional file 1: Table S4). Of note, we observed significant gender differences (p = 0.01) among the C3 community type (Additional file 1: Fig. S4a). Tumor locations (colon or rectum; p = 0.01) were linked to the C4 community type, which primarily consisting of the beneficial species (Additional file 1: Fig. S4b).

### Gene mutation profile and microbiota composition and functional features

**Fig. 3:**
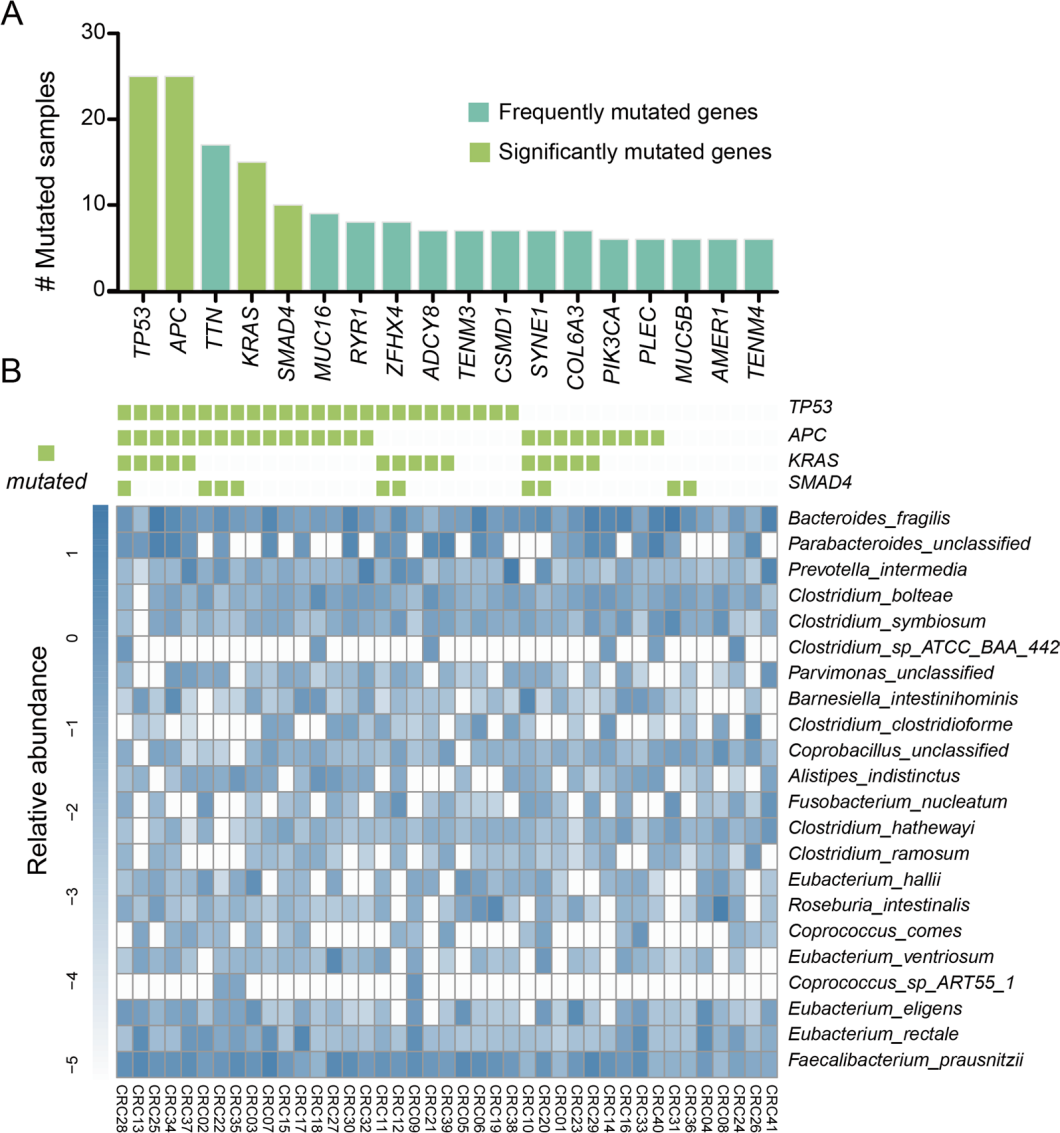
An overview of the associations between cancer genome and microbiome genomes. **A** Bar plots illustrate the frequently mutated genes in 41 tumor tissues. **B** The interaction between gut microbial taxa and somatic altered genes

Previous studies indicated that gut microbes may induce DNA damage, thereby accelerating cancer development [29]. Consequently, we detected somatic mutations using exome sequencing technology from 41 CRC tumors and idntified 4 significantly mutated genes with MutSigCV, including *TP53* (Q value = 0), *APC* (Q value = 1.26E-11), *KRAS* (Q value = 1.11E-10) and *SMAD4* (Q value = 7.37E-04) (Fig. 3A and Additional file 1: Table S6).

**Fig. 4:**
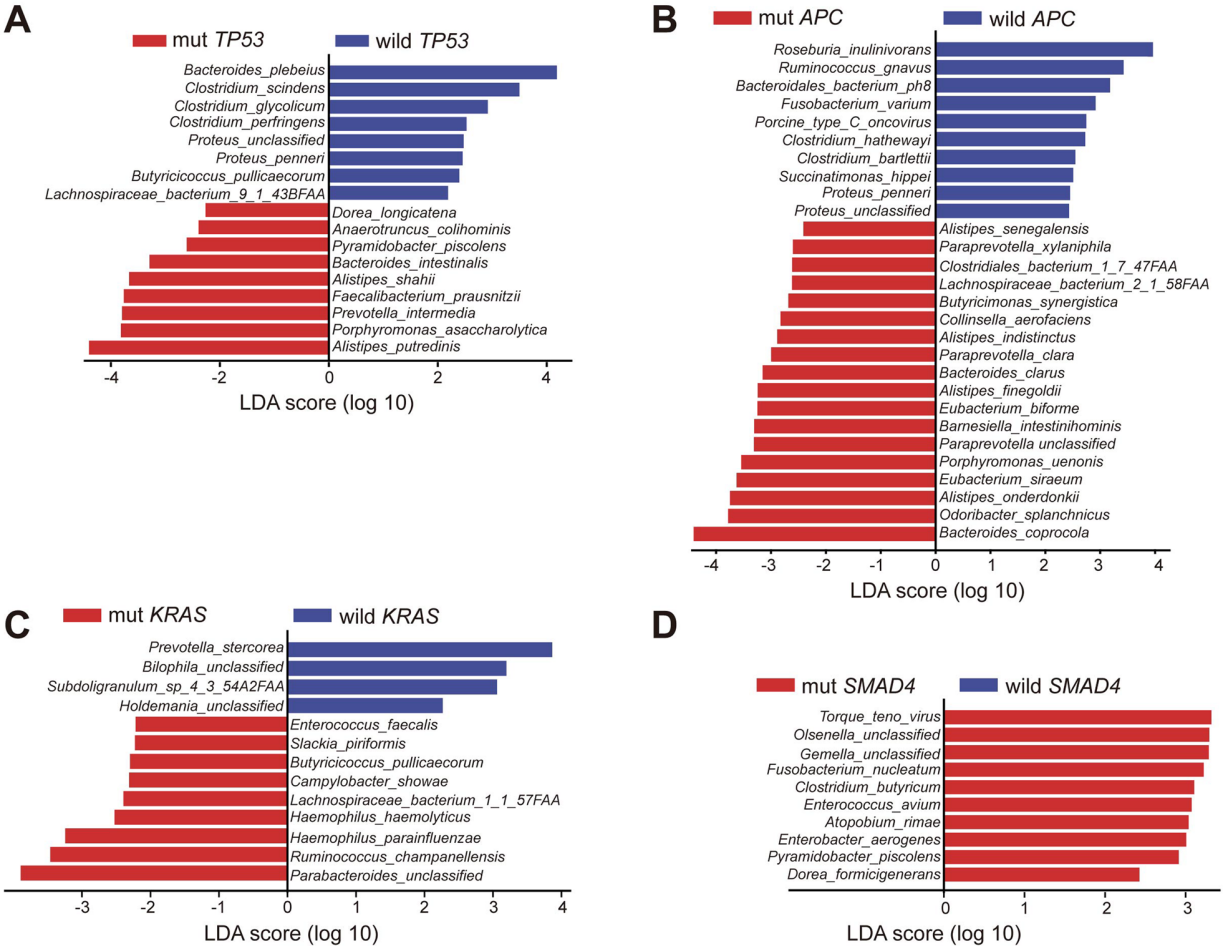
**A**–**D** Significantly mutated genes related taxonomic difference. Differentially abundant species between tumors with and without *TP53* (**A**), *APC* (**B**), *KRAS* (**C**), *SMAD4* (**D**) alterations, respectively

To explore their associations with microbiota composition, we conducted the LEfSe analysis to compare tumors with or without mutated genes (Fig. 3B). *TP53* is the most prevalent somatic altered genes in our cohort. In *TP53* mutated subjects, an enrichment of several disease-associated species, including *Alistipes putredinis* (LDA score = 4.402)*, Porphyromonas asaccharolytica* (LDA score = 3.816), and *Prevotella intermedia* (LDA score = 3.795) (Fig. 4A). Previous observations uncovered that butyrate treatment could activate the *TP53* pathway [30]. Consistently, the abundance of butyrate-producing bacteria, *Butyricicoccus pullicaecorum*, exhibited a significant reduction in *TP53* mutation carriers (LDA score = 2.395). Interestingly, *Roseburia inulinivorans* (LDA score = 3.96) and *Ruminococcus gnavus* (LDA score = 3.426)*,* two other butyrate producers, were also significantly depleted in *APC* mutation carriers (Fig. 4B). Besides, the relative abundance of *Enterococcus* genus was enriched in subjects with *KRAS* and *SMAD4* mutations (*Enterococcus faecalis*, LDA = 2.217; *Enterococcus avium,* LDA score = 3.075) (Fig. 4C, D). We also performed similar analysis between gut microbiota and other frequently mutated genes (Additional file 1: Fig. S5). In stool samples, probiotics, including *Ruminococcus lactaris* (LDA score = 3.405), *Bifidobacterium bifidum* (LDA score = 2.425), were dramatically elevated in MUC5B or MUC16 mutated individuals (Additional file 1: Fig. S5e, f). *Barnesiella intestinihominis,* acting as an enhancer for anticancer therapy, was proven enriched in *TNN* mutation carriers (LDA score = 3.156) (Additional file 1: Fig. S5m).

We further characterized the differences of microbial pathways between subjects with specific mutations and control group. Interestingly, the most abundant pathways were generally housekeeping processes encoded by microbes, such as one carbon metabolism, aromatic amino acids, branched chain amino acid and so on (Additional file 1: Fig. S6). One-carbon (1C) metabolism, consistently overexpressed in cancer, supports multiple biological processes, including nucleotides synthesis, methionine recycling pathway and redox defense [31]. An increased level of bacterial purine (reporter score = 2.909) and pyrimidine (reporter score = 3.188) metabolism were found in *TP53* mutation carriers (Additional file 1: Fig. S6a). Similarly, bacterial cysteine-methionine metabolism (reporter score = 3.246) and folate biosynthesis (reporter score = 1.949) exhibited significant alterations in individuals with *APC* mutations (Additional file 1: Fig. S6b). Bacteria can synthesize different amino acids. Compared to control group, we found *APC* (Additional file 1: Fig. S6c) and *SMAD4* mutation carriers (Additional file 1: Fig. S6d) were significantly associated with high levels of bacterial tryptophan (Trp) metabolism pathway (reporter score = 3.045 and 2.732, respectively). Moreover, we observed an elevated abundance of bacterial phenylalanine metabolism correlated with *KRAS* mutations (reporter score = 4.345) (Additional file 1: Fig. S6c).

### Gene expression signature and metabolic pathways reprogramming associated with microbial shifts

**Fig. 5:**
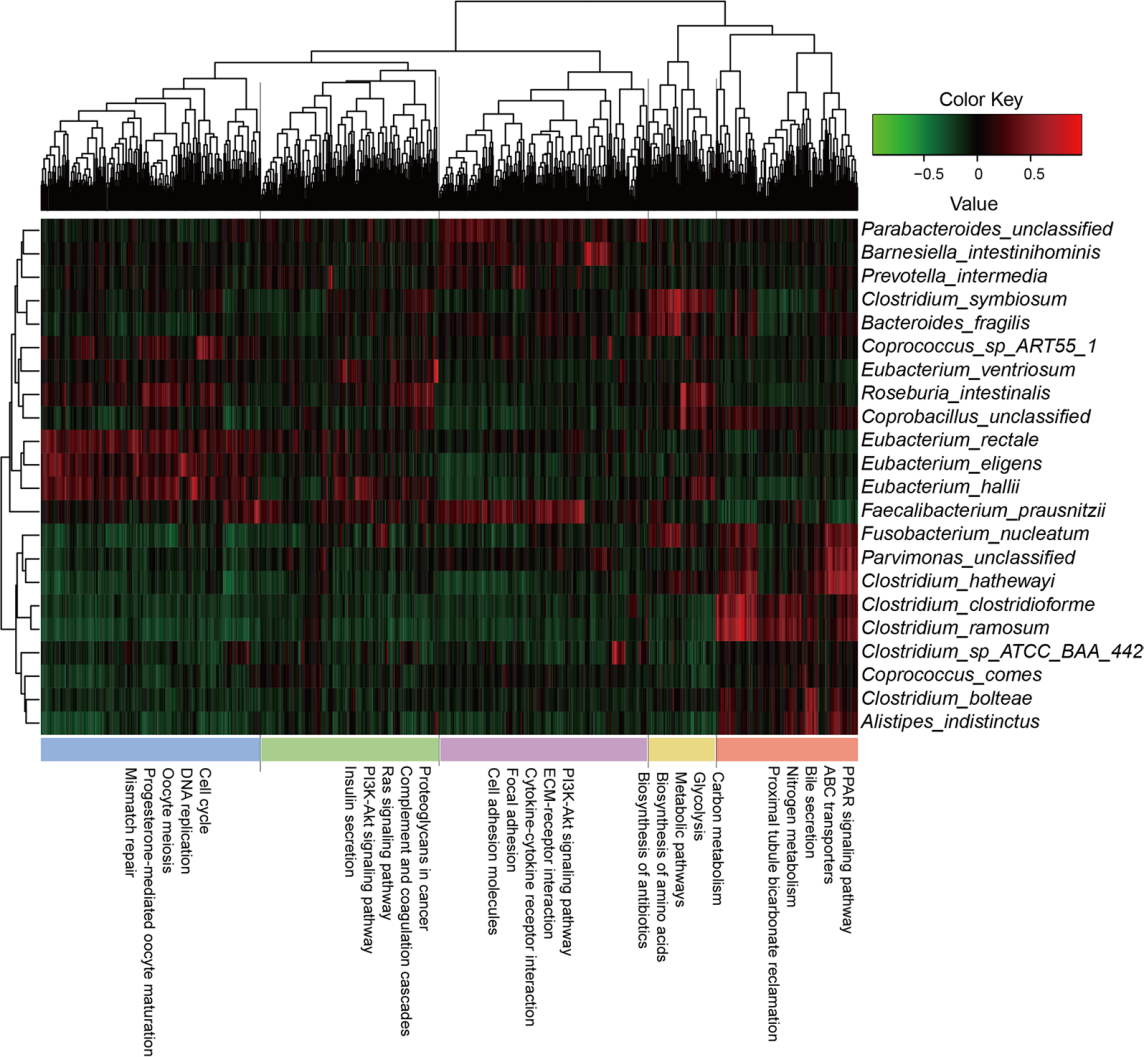
Correlation of differentially abundant species and deregulated genes. Tumor associated deregulated genes were clustered and annotated with DAVID. The X axis illustrated the DAVID functional annotation and Y axis showed differentially abundant species. Red color represents positive association while green color means negative association

We also investigated the relationship between the microbiome composition and the gene expression patterns in CRC. We observed that certain bacterial species were significantly correlated with the gene expression pattern (Additional file 1: Fig. S7 and Table S6). The differentially expressed functional genes were clustered according to their correlation with differentially abundant species, following by annotation with DAVID (Fig. 5). We observed that *Fusobacterium nucleatum*, along with some *Clostridium spp.* exhibited positive associations nitrogen metabolism and bile secretion pathways, but negatively with cytokine-cytokine receptor interaction pathway.

**Fig. 6:**
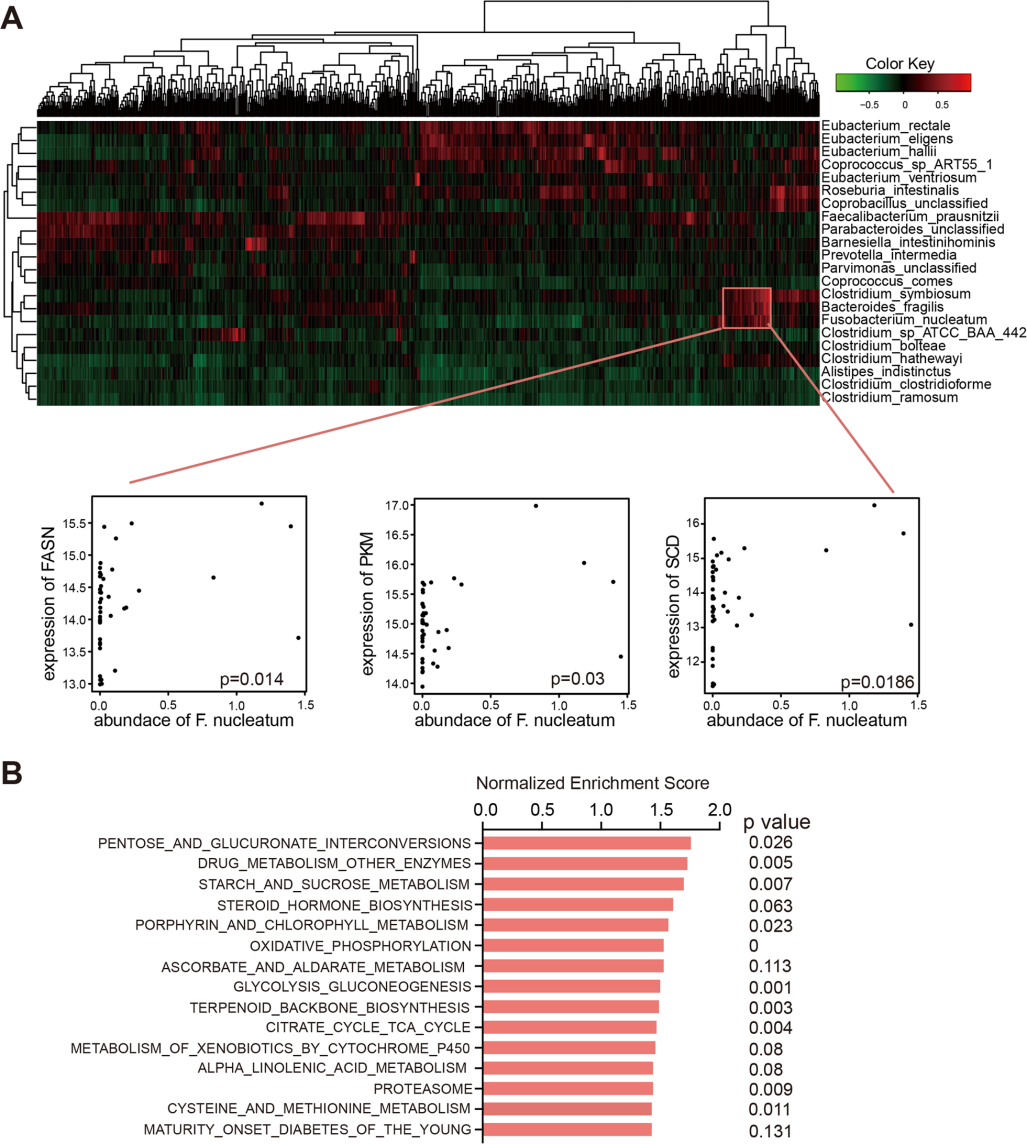
Gene expression signature and metabolic pathways reprogramming associated with microbial shifts. **A** The association between up regulated oncogene expression and cancer related species. The X axis represents up regulated cancer genes. Significant associations were highlighted below the heatmap. **B** Pathway difference between high and low *Fusobacterium nucleatum* groups

Subsequently, the interaction between 22 bacterial species and up-regulated oncogene expression was explored. As shown in Fig. 6A, *Fusobacterium nucleatum* was positively correlated with *PKM* (p = 0.03), *SCD* (p = 0.0186), *FASN* (p = 0.014), which are key enzymes in glycolysis and fatty acid metabolism. Consistent with the findings, we categorized patients into high and low *Fusobacterium nucleatum* groups, and found that various metabolism related pathways were significantly enriched in the high groups (pentose and glucuronate interconversions, p = 0.026; starch and sucrose metabolism, p = 0.007; porphyrin and chlorophyll metabolism, p = 0.023; oxidative phosphorylation, p < 0.00001) (Fig. 6B). Taken together, the intestinal microbiota promotes CRC progression by shaping the expression of host gene expression, especially metabolic pathways.

### *Fusobacterium nucleatum* promoted CRC by modifying the tumor immune environment and* TNFSF9* expression

**Fig. 7:**
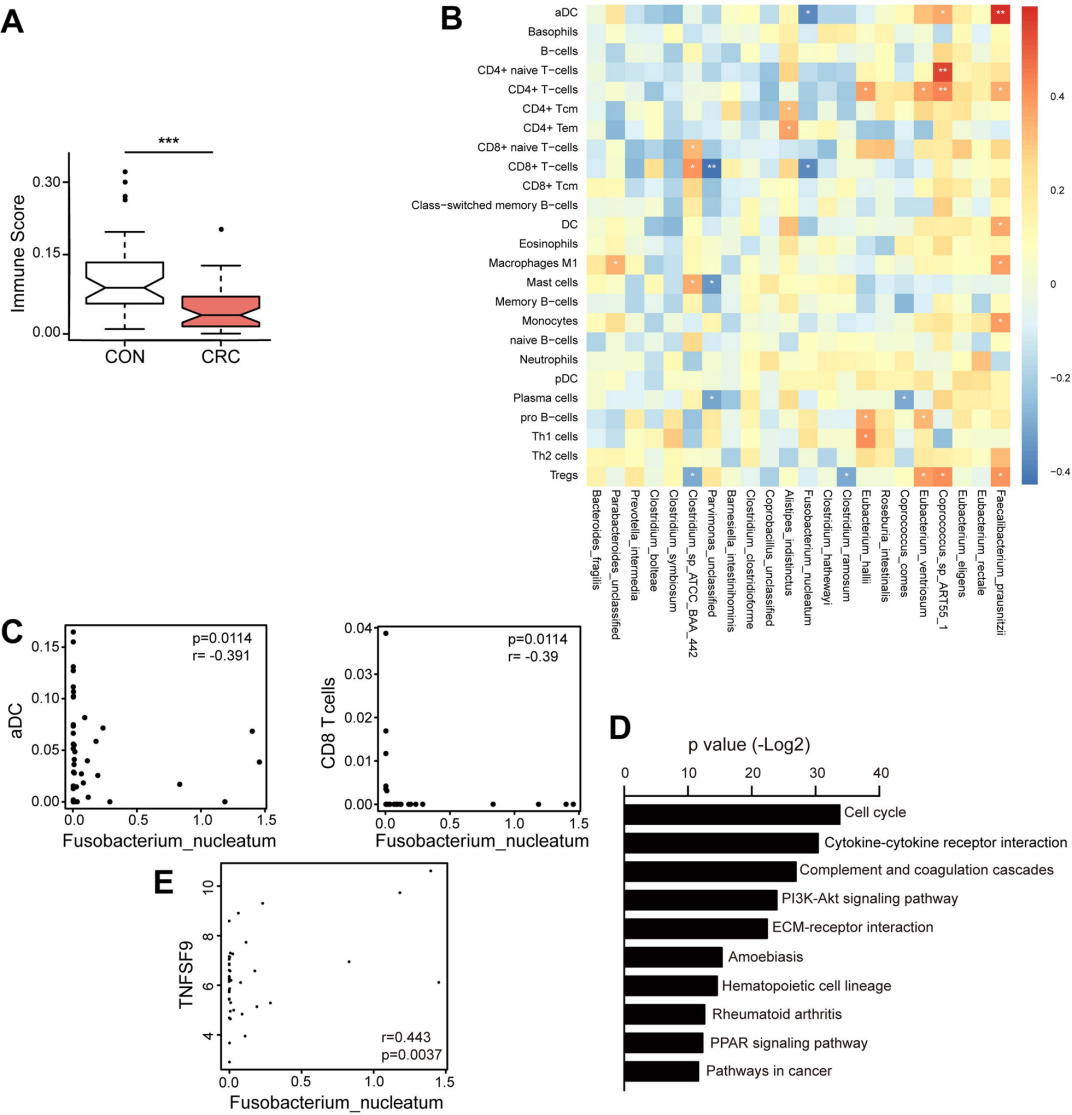
*Fusobacterium nucleatum* promoted CRC by modifying the tumor immune environment and *TNFSF9* expression. **A** Comparison of immune cell scores between tumor and adjacent normal tissues. **B** The heatmap illustrates the correlations between differential abundant species and immune cells. The stars indicate the level of statistical significance. **C** Significant association of *F. nucleatum* and aDC and CD8 T cells. **D** Pathway alteration between normal and tumor tissue. **E** Significant association between *Fusobacterium nucleatum* and *TNFSF9* gene expression

The composition of immune and stromal cell types was identified by XCELL, a gene signature-based method that integrates the advantages of gene set enrichment with deconvolution approaches. Compared with adjacent normal tissues, the overall immune score was significantly lower in tumor tissue (Fig. 7A). Especially, the abundance of most B cells and CD8 + T cells elevated in tumors while regulatory T cells and T helper cells exhibited a decreasing trend (Additional file 1: Fig. S8), indicating the important role of the immune microenvironment in the progression of CRC. The associations between different microbial species and immune cell types in the CRC were shown in Fig. 7B. *Fusobacterium nucleatum* was negatively associated with dendritic cells and CD8 T cells (Fig. 7C). While *Faecalibatcerium prausnitzii* were significantly positively correlated with dendritic cells and Macrophages M1 (Additional file 1: Fig. S9a).

Interestingly, Gene set enrichment analysis of revealed that the cytokine-cytokine receptor interaction (*p* < *0.001*) was significantly altered in CRC (Fig. 7D). Correlation analysis of genes related to cytokine-cytokine receptor interaction pathway related genes and 22 species uncovered several significant associations (Additional file 1: Fig. S9b). Among them, *Fusobacterium nucleatum* exhibited a positive association with *TNFSF9*, a member of TNF (tumor necrosis factor) family members (*r* = *0.443, p* = *0.0037*) (Fig. 7E). Previous study showed that *Fusobacterium nucleatum* autoinducer-2 (AI-2) enhanced the mobility and M1 polarization of macrophages, possibly through TNFSF9/TRAF1/p-AKT/IL-1β signaling. Our results further suggested that pathogenic bacteria, like *Fusobacterium nucleatum,* may interact with CRC cells and modify the tumor immune environment by TNFSF9, finally facilitating the tumor development.

## Discussion

Microbiota studies of stool samples from CRC patients have shown that certain *Bacteroides* spp., including *B.* *dorei, B. vulgatus*, and *B.* *massiliensis*, and *E. coli*, were correlated with tumor stage [32]. We have characterized 22 bacterial strains associated with CRC in Chinese population. Although we have depicted the diversity of the gut microbiota in CRC, the features of most bacterial species in CRC remains largely unknown. The complexity of the human intestinal microbiota, with a plethora of uncharacterized host-microbe, microbe-microbe, and environmental interactions, presents a challenge of advancing our knowledge of the intestinal microbiota-cancer interaction. In this study, a number of bacterial metabolism associated pathways, such as one carbon pool by folate and oxidative phosphorylation were found activated in CRC groups. It’s of great interest for us to explore how bacteria interact with host through metabolites in the future.

CRC is considered a disease associated with the accumulation of genetic alterations during tumorigenesis. Recently, human gut microbiota has been shown to have pivotal roles in contributing to the development of CRC. Little is known regarding microbiome-gene interactions during CRC tumorigenesis. It has been shown that host genetics can influence the abundance of microbial taxa, as demonstrated in the studies of monozygotic twins [33]. We demonstrated that certain bacterial species are particularly affected by specific gene mutations, including *TP53*, *KRAS*, *APC*, *SMAD4,* and so on. We hypothesize that epithelial cells in the colon under a mutated status are compensated by species that take advantage of this new microenvironment. Therefore, certain bacteria could be manifested during colon tumorigenesis. In addition, we also figured out the bacterial KEGG pathways that were enriched in different mutation carriers. Notably, metabolic pathways came out. However, the correlation between different mutations, CRC-associated taxa and its pathways need further investigation.

Some CRC cases are associated with inflammation, which is one of the hallmarks of cancer. Inflammatory mechanisms are critical drivers of tumorigenesis, which has also been observed in a portion of CRC patients with inflammatory bowel disease. The microbiota is critical in shaping an inflammatory microenvironment, which in turn affects the microbial composition. Carcinogenesis in the intestine due to gene dysregulation can affect the presence of microbes, inflammation, and the modulation of intestinal immunity, as demonstrated by the interplay between a defective gene status and microbial composition. We found that the cytokine-cytokine receptor interaction and complement and coagulation cascades related genes of host are regulated in CRC. Previous studies have demonstrated that metabolism could fuel the immune system [34, 35]. Consistently, we showed the interplay between bacteria and metabolism related pathways of host. Since bacteria also have metabolic systems, we speculate the metabolites produced by bacteria may crosslink with CRC patient metabolim, leading to the immune response. Further investigation for the elucidation of the mechanisms needs to be performed.

Microbial biomarkers are already recognized as an independent factor in cancer. Given that CRC-associated taxa can impact inflammatory pathways and metabolism, it is possible that targeting the gut microbiota may be effective to improve the clinical diagnostic accuracy and efficacy [36]. Microbiota such as *Fusobacterium nucleatum* is highly enriched in CRC tissues and fecal, and consequently is an excellent diagnostic marker for early diagnosis and prognosis prediction of CRC [37]. The convinced crosstalk of microbiota (*Fusobacterium nucleatum*) to host functional pathways indicating that the microbiota also a promising therapy target to treat CRC, though further studies are needed to investigate its functional impact in CRC and the underlying mechanism.

### Supplementary Information

**Additional file 1: Fig. S1.** **a** Sequencing information for exome sequencing, transcriptome sequencing and metagenomic sequencing data, respectively. **b** Comparison of shannon index across CRC-cohort1, healthy controls and CRC-cohort2. **c** Comparison of Bray-Curtis distances across CRC-cohort1, healthy controls and CRC-cohort2. **Fig. S2.** The blue nodes represent species depleted in cancer group while orange nodes represent enriched species. The green nodes represent metabolic pathways. The blue and orange lines represent negative and positive correlations, respectively. **Fig. S3.** Bar plots illustrated AGE (**a**), GENDER (**b**), LOCATION (**c**) and STAGE (**d**) associated taxonomy difference. **Fig. S4.** Box plots showed significant association between species’s clusters and clinic elements, such as GENDER (**a**), Location (**b**). **Fig. S5.** Bar plots illustrated somatic mutated genes associated taxonomy difference. **Fig. S6.** Bar plots illustrated somatic mutated genes associated pathway difference. **Fig. S7.** The overview of interactions between cancer associated deregulated genes and differentially abundant species. The X axis represents the deregulated genes and Y axis showed differentially abundant species. Red color represents positive association while green color means negative association. **Fig. S8.** Illustration of lymphoid and myceloid immune cells changes between tumor and adjacent normal tissues. **Fig. S9.** **a** Correlation of *F. prausnitzii* and aDC and Macrophages M1 cells. **b** Association of bacteria and host genes on cytokine-cytokine receptor pathway

## Data Availability

All data generated or analysed during this study are available from the corresponding author upon reasonable request.
