# Supplementary material for: Multi-omic profiling reveals associations between the gut microbiome, host genome and transcriptome in patients with colorectal cancer
Source: J Transl Med. 2024 Feb 18;22:175. doi: 10.1186/s12967-024-04984-4 (PMC10874565; doi:10.1186/s12967-024-04984-4)
Supplement: Supplementary file 1 — Additional file 1: Fig. S1. a Sequencing information for exome sequencing, transcriptome sequencing and metagenomic sequencing data, respectively. b Comparison of shannon index across CRC-cohort1, healthy controls and CRC-cohort2. c Comparison of Bray-Curtis distances across CRC-cohort1, healthy controls and CRC-cohort2. Fig. S2. The blue nodes represent species depleted in cancer group while orange nodes represent enriched species. The green nodes represent metabolic pathways. The blue and orange lines represent negative and positive correlations, respectively. Fig. S3. Bar plots illustrated AGE (a), GENDER (b), LOCATION (c) and STAGE (d) associated taxonomy difference. Fig. S4. Box plots showed significant association between species’s clusters and clinic elements, such as GENDER (a), Location (b). Fig. S5. Bar plots illustrated somatic mutated genes associated taxonomy difference. Fig. S6. Bar plots illustrated somatic mutated genes associated pathway difference. Fig. S7. The overview of interactions between cancer associated deregulated genes and differentially abundant species. The X axis represents the deregulated genes and Y axis showed differentially abundant species. Red color represents positive association while green color means negative association. Fig. S8. Illustration of lymphoid and myceloid immune cells changes between tumor and adjacent normal tissues. Fig. S9. a Correlation of F. prausnitzii and aDC and Macrophages M1 cells. b Association of bacteria and host genes on cytokine-cytokine receptor pathway [file 12967_2024_4984_MOESM1_ESM.docx]

**Additional file 1**

**Multi-omic profiling reveals associations between the gut microbiome, host genome and transcriptome in patients with colorectal cancer**

Shaomin Zou^1,2,3^*, Chao Yang^4^*, Jieping Zhang^1,2,3^*, Dan Zhong^1,2,3^, Manqi Meng^1,2,3^, Lu Zhang^4^, Honglei Chen^5#^ and Lekun Fang^1,2,3#^

**
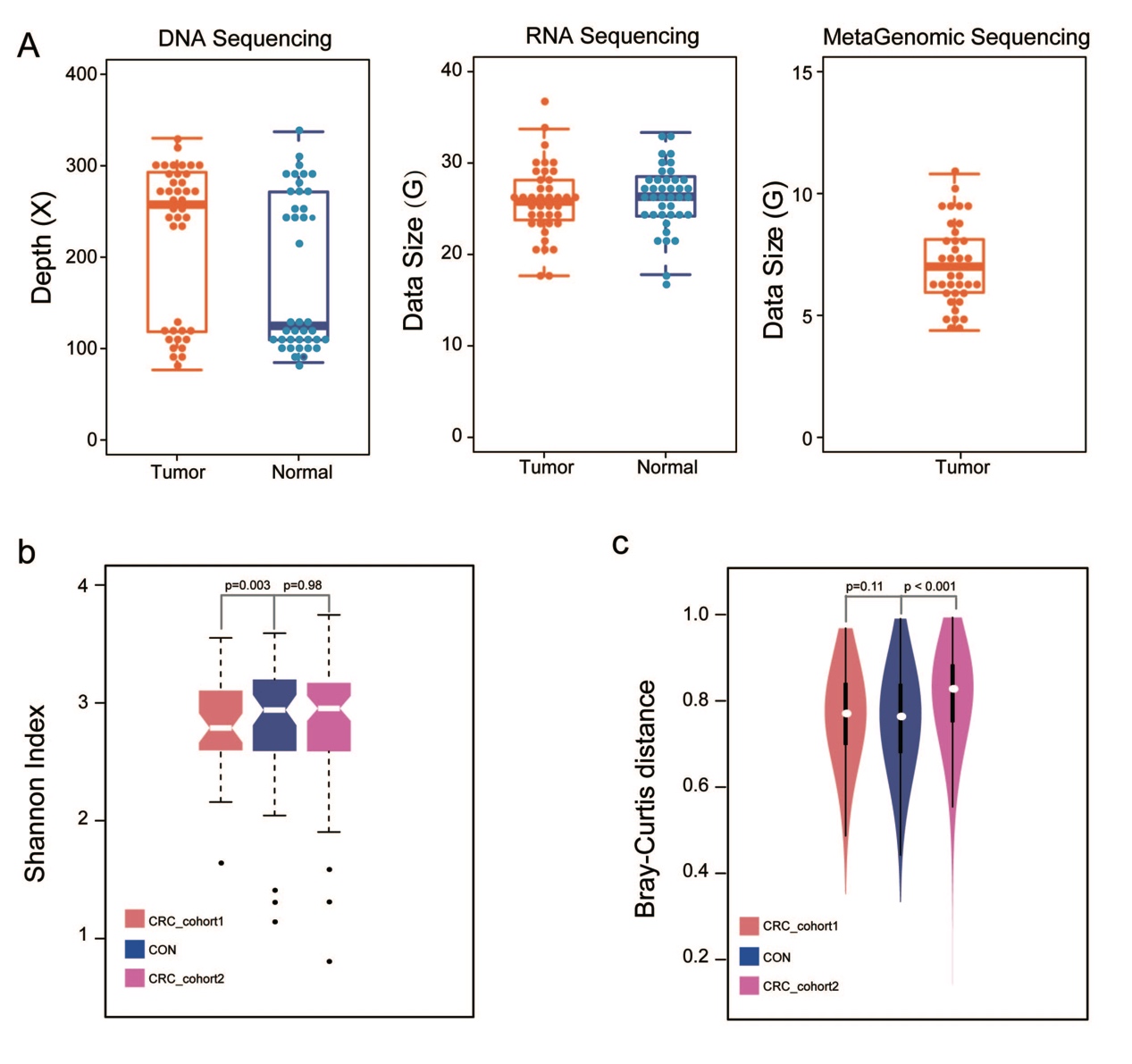
Fig. S1 a** Sequencing information for exome sequencing, transcriptome sequencing and metagenomic sequencing data, respectively. **b** Comparison of shannon index across CRC-cohort1, healthy controls and CRC-cohort2. **c** Comparison of Bray-Curtis distances across CRC-cohort1, healthy controls and CRC-cohort2.


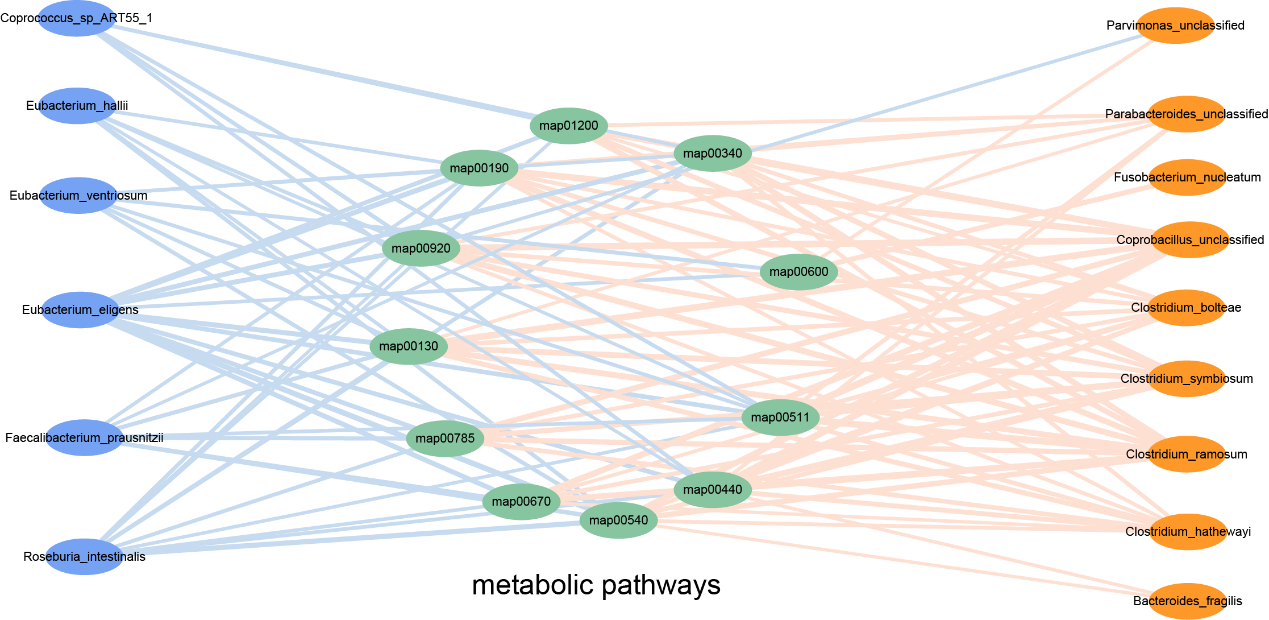


**Fig. S2** The blue nodes represent species depleted in cancer group while orange nodes represent enriched species. The green nodes represent metabolic pathways. The blue and orange lines represent negative and positive correlations, respectively.


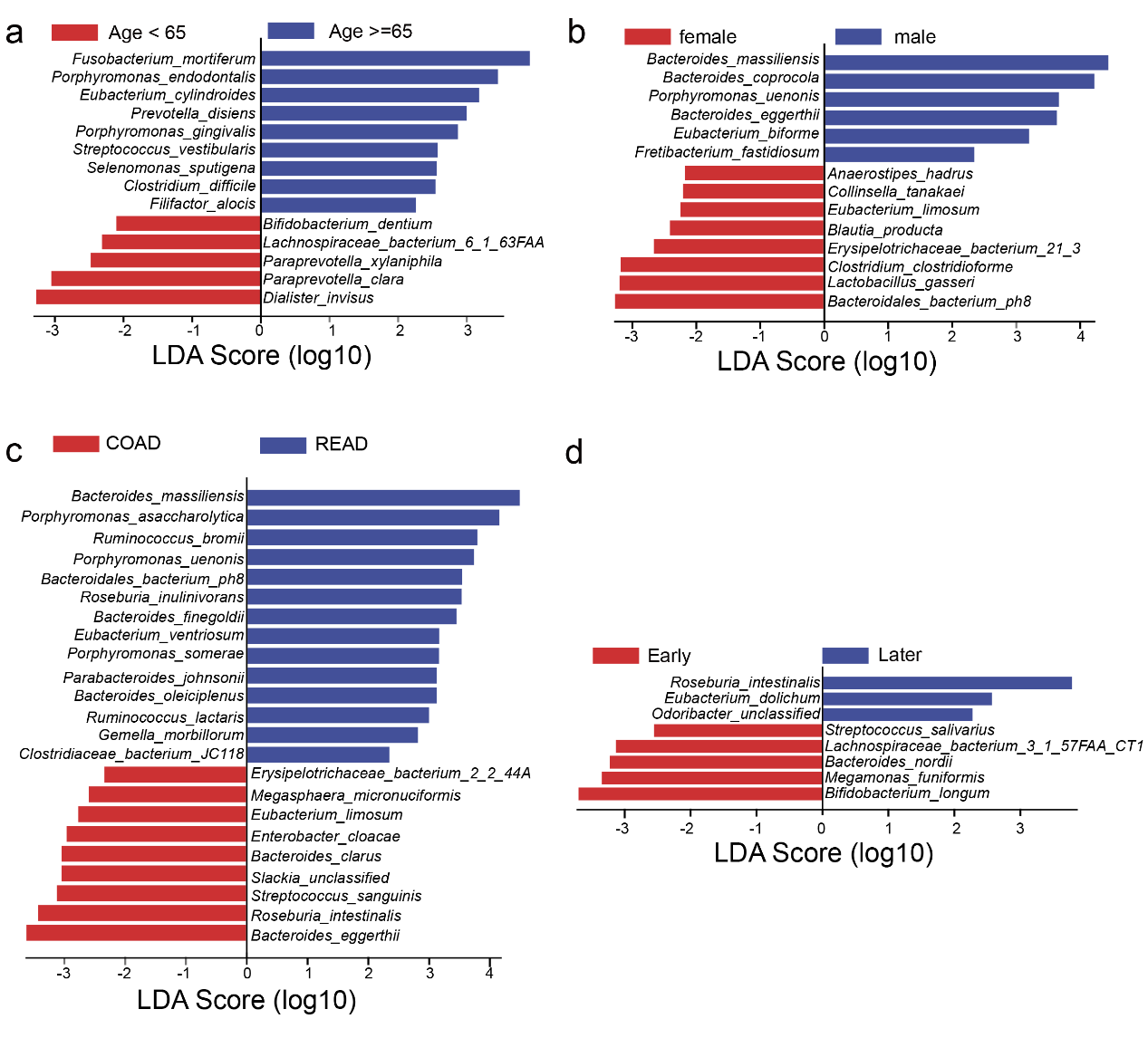


**Fig. S3** Bar plots illustrated AGE(**a**), GENDER(**b**), LOCATION(**c**) and STAGE(**d**) associated taxonomy difference.


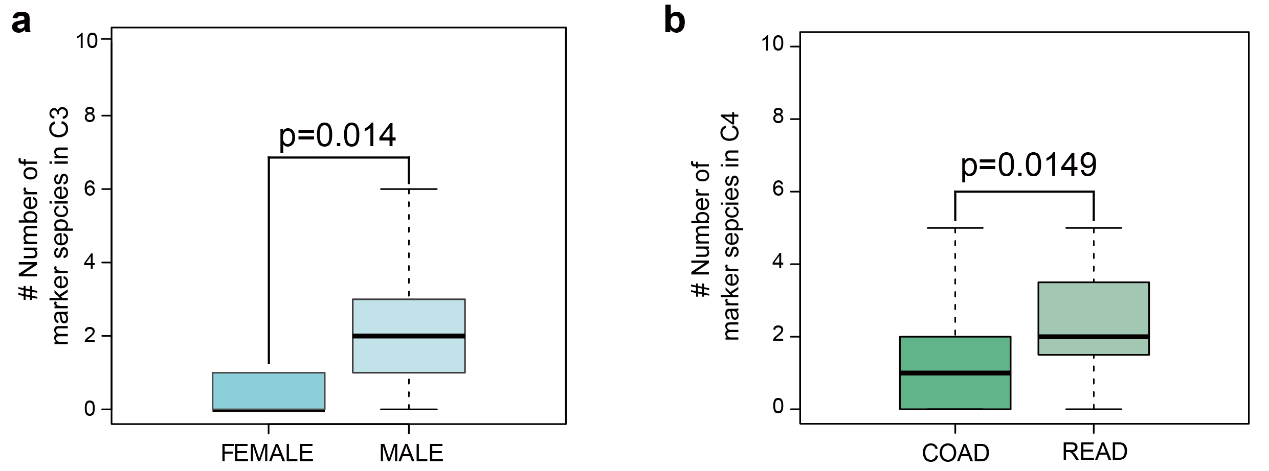


**Fig. S4** Box plots showed significant association between species’s clusters and clinic elements, such as GENDER(**a**), Location(**b**).


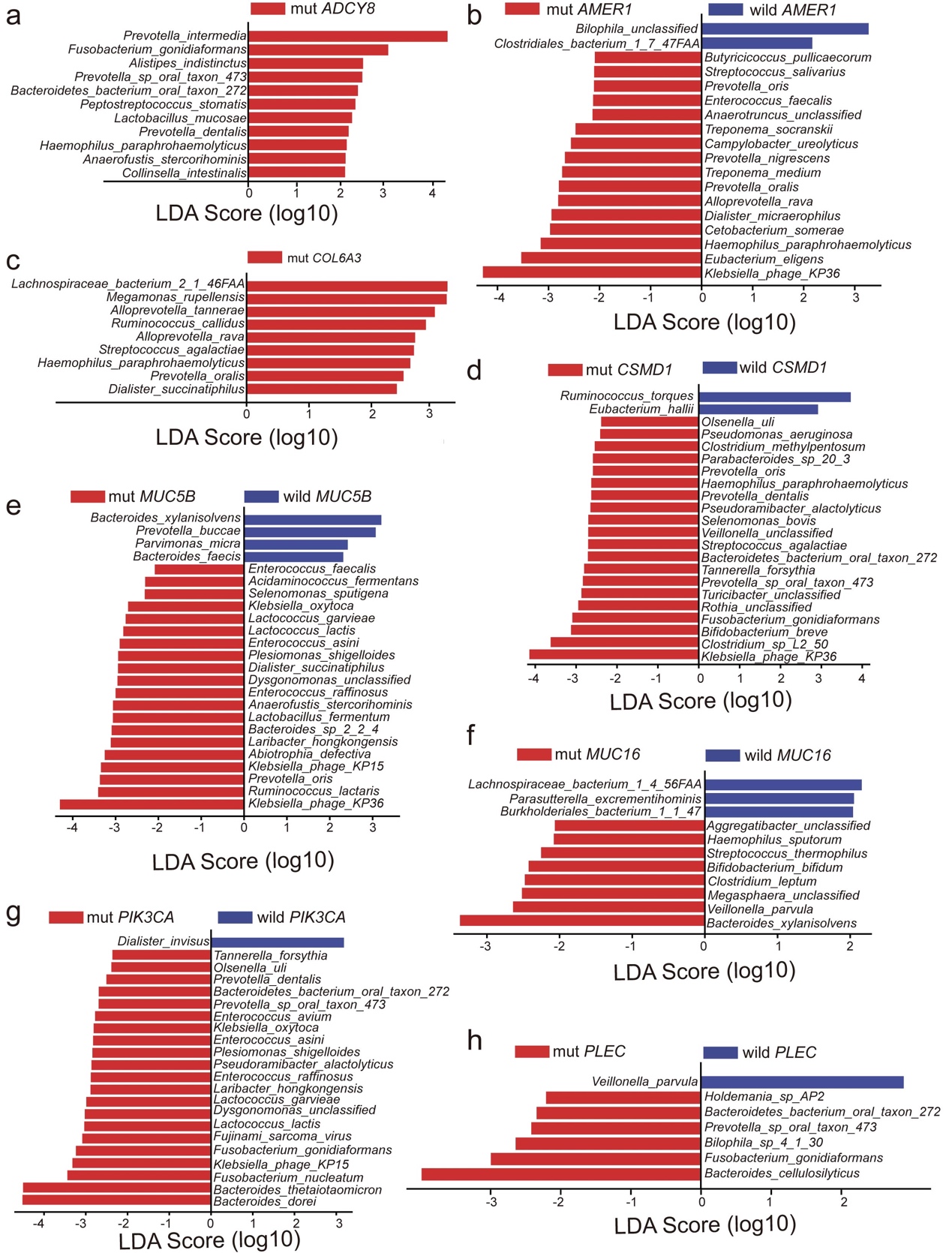


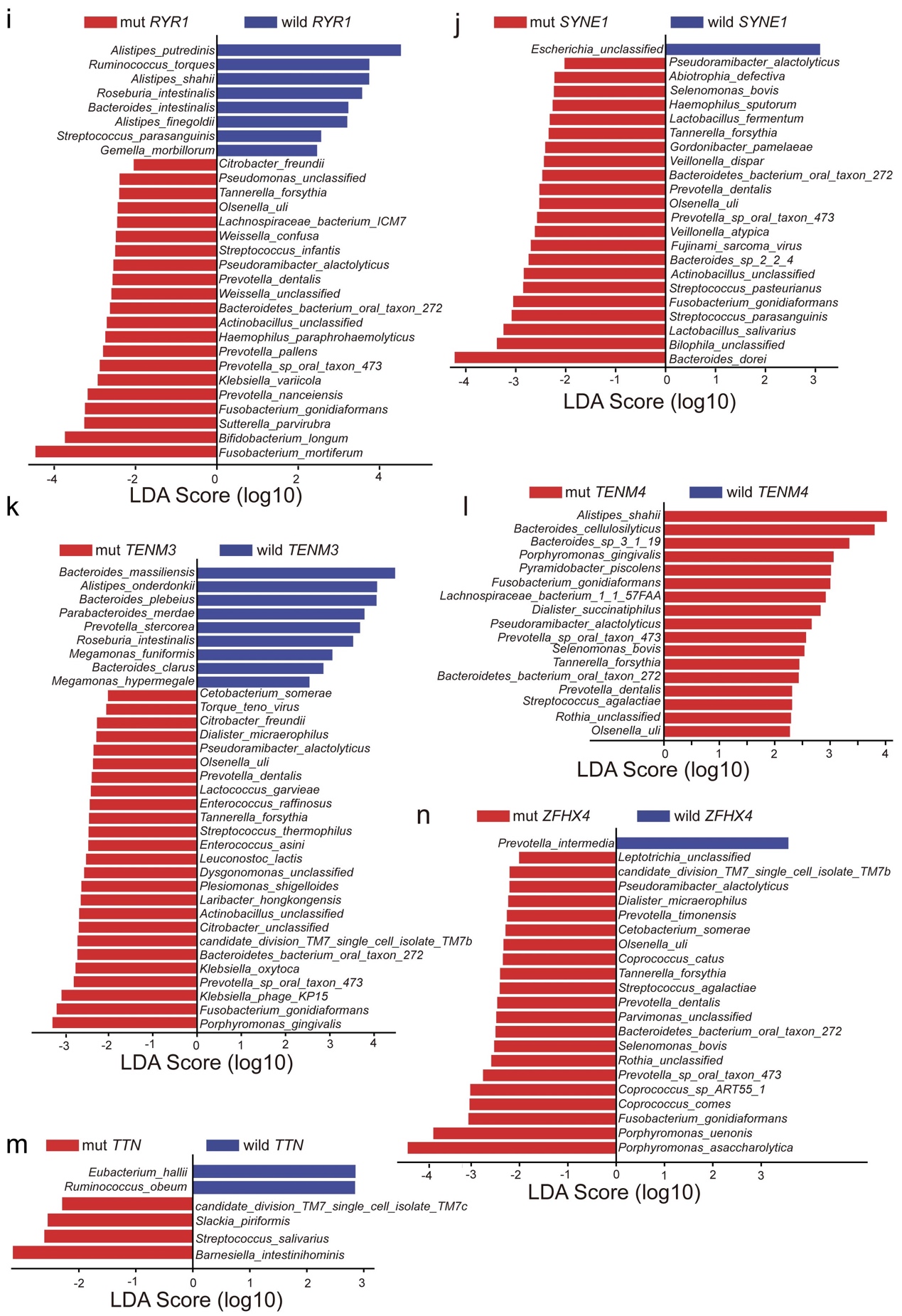


**Fig. S5** Bar plots illustrated somatic mutated genes associated taxonomy difference.


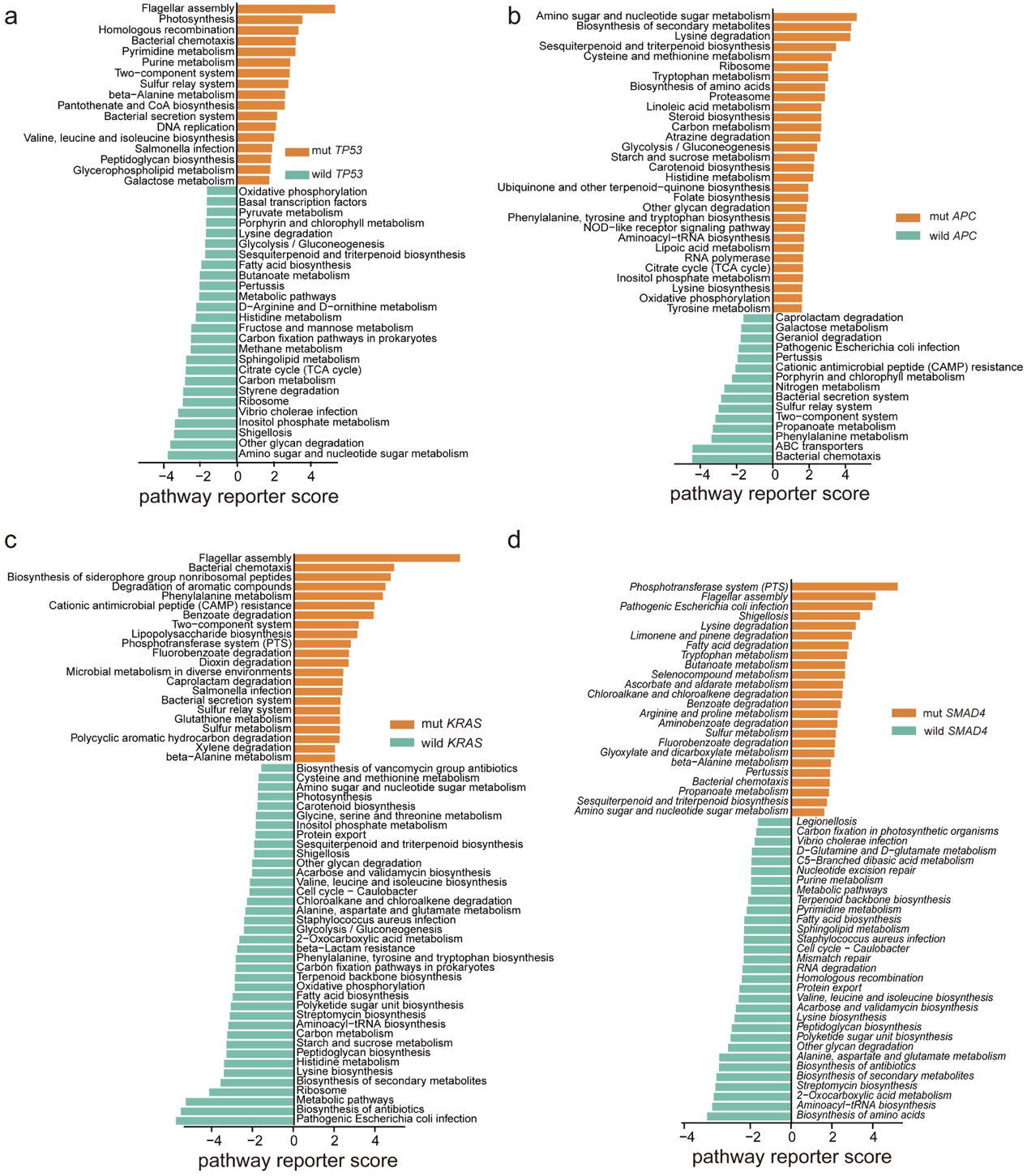


**Fig. S6** Bar plots illustrated somatic mutated genes associated pathway difference.


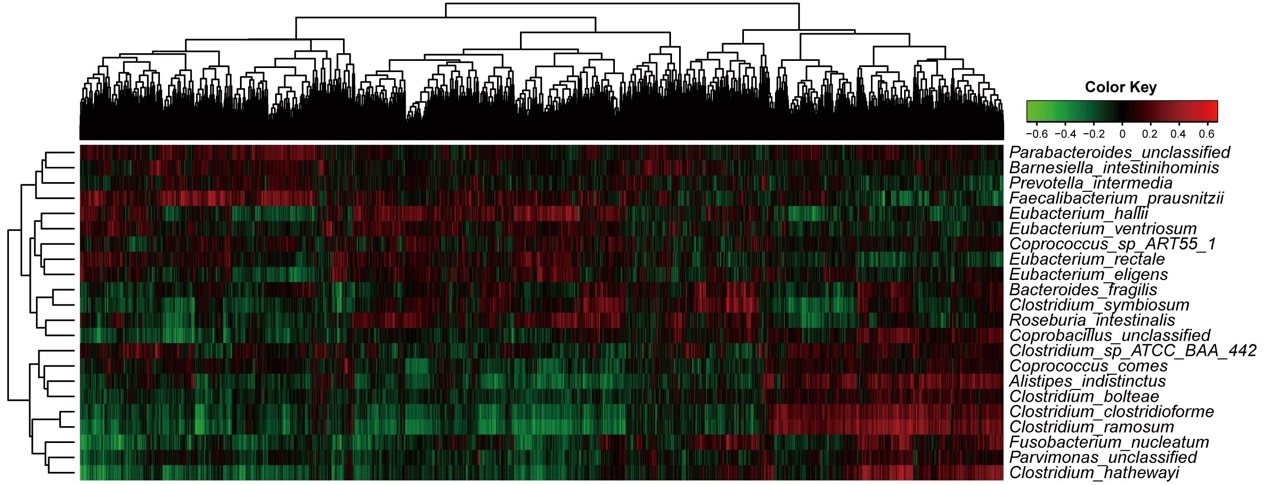


**Fig. S7** The overview of interactions between cancer associated deregulated genes and differentially abundant species. The X axis represents the deregulated genes and Y axis showed differentially abundant species. Red color represents positive association while green color means negative association.


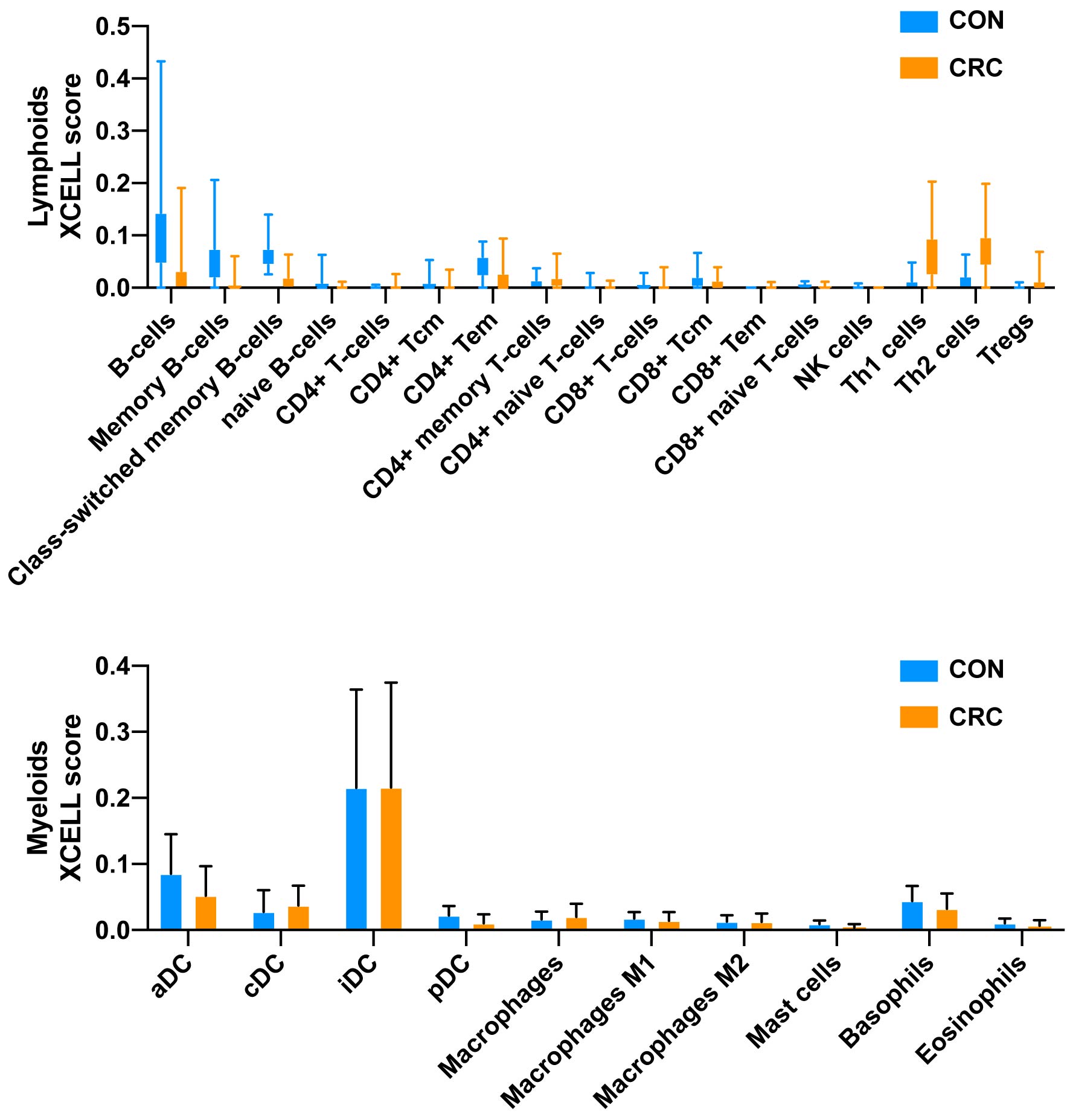


**Fig. S8** Illustration of lymphoid and myceloid immune cells changes between tumor and adjacent normal tissues.


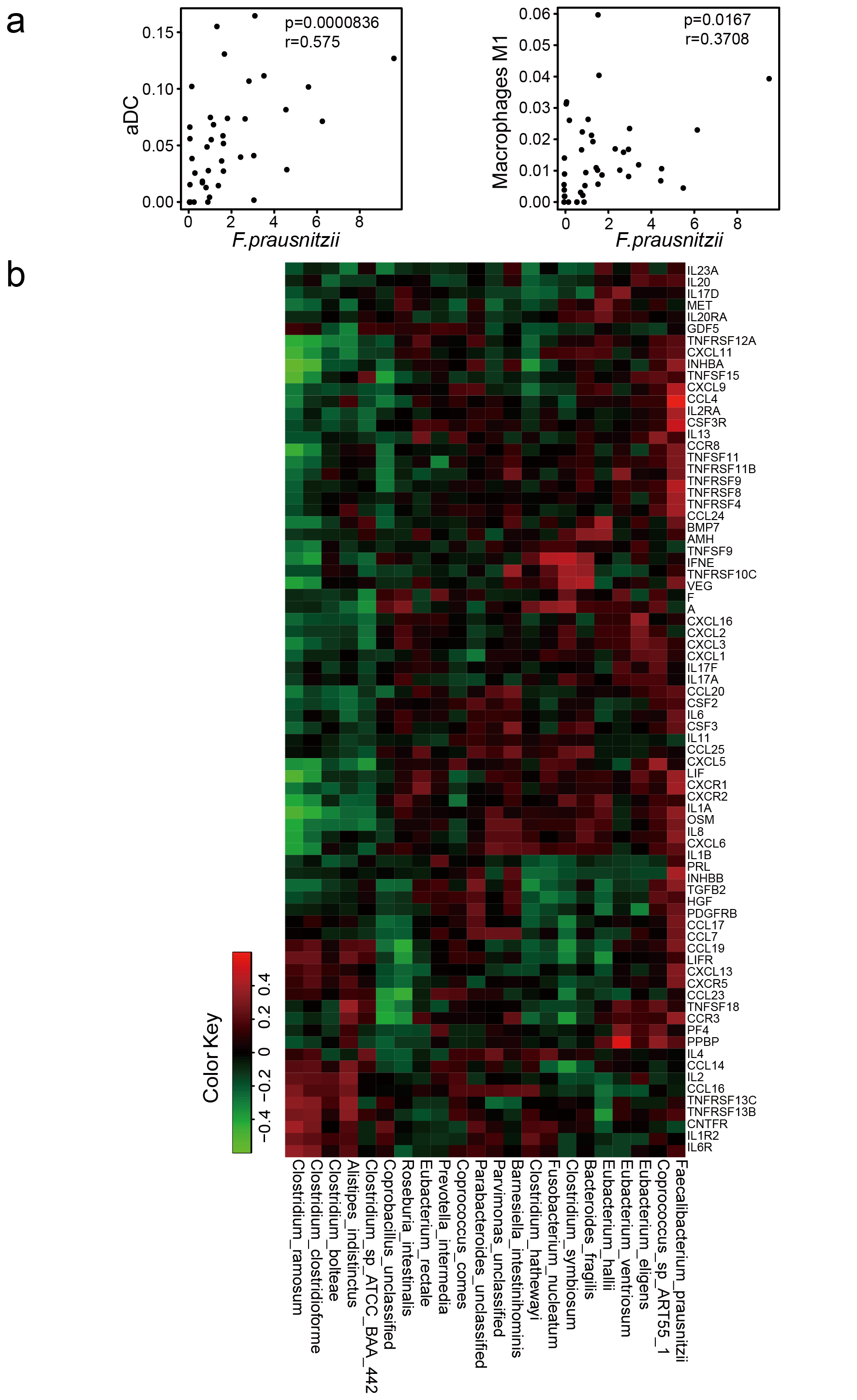


**Fig. S9** **a** Correlation of *F. prausnitzii* and aDC and Macrophages M1 cells. **b** Association of bacteria and host genes on cytokine-cytokine receptor pathway
